# Inter-relationship of plasma markers of oxidative stress and thyroid hormones in schizophrenics

**DOI:** 10.1186/1756-0500-5-169

**Published:** 2012-03-31

**Authors:** Moses O Akiibinu, Omobola A Ogundahunsi, Ebenezer O Ogunyemi

**Affiliations:** 1 Department of Chemical Pathology and Immunology, College of Health Sciences, Olabisi Onabanjo University, Ago-Iwoye, Ogun State, Nigeria; 2 G.P.O Box 11379 Dugbe, Ibadan, Oyo State, Nigeria

**Keywords:** Oxidative stress, Thyroid hormones, Schizophrenia

## Abstract

**Background:**

The relationship of oxidative stress to thyroid hormones has not been studied in the schizophrenics. The present study determined the status and interrelationship of plasma markers of oxidative stress, nitric oxide and thyroid hormones in thirty (17 males and 13 females) newly diagnosed patients with acute schizophrenia before initiation of chemotherapy. Twenty five (13 males and 12 females) mentally healthy individuals served as controls. Patients and controls with history of hard drugs (including alcohol and cigarette), pre-diagnosis medications (e.g. antiparkinsonian/antipsychotic drugs), chronic infections, liver disease and diabetes mellitus were excluded from the study. Plasma levels of total antioxidant potential (TAP), total plasma peroxides (TPP), nitric oxide (NO), malondialdehyde (MDA), thyroxine (T4), tri-iodothyronine (T3) and thyroid stimulating hormone (TSH) were determined in all participants using spectrophotometric and enzyme linked immunosorbent assay (ELISA) methods respectively. Oxidative stress index (OSI) was calculated as the percent ratio of total plasma peroxides and total antioxidant potential.

**Findings:**

Significantly higher plasma levels of MDA (p < 0.01), TPP (p < 0.01), OSI (p < 0.01), T3 (p < 0.01) and T4 (p < 0.05) were observed in schizophrenics when compared with the controls. The mean levels of TAP, NO and TSH were significantly lower in schizophrenics (p < 0.01) when compared with the controls. The result shows that T3 values correlate significantly with MDA (p < 0.05) and TPP (p < 0.01) in schizophrenics.

**Conclusions:**

Higher level of TPP may enhance thyroid hormogenesis in schizophrenics. Adjuvant antioxidant therapy may be a novel approach in the treatment of schizophrenic patients.

## Introduction

Schizophrenia is a disorder of aberrant neurodevelopment with minor physical anomalies, neurological soft signs, and abnormalities of brain structure and function [1]. The brain abnormalities therefore cause deficits in both working memory and long term memory tasks [2]. It is marked by disturbances in thinking, emotional reaction, social behavior, with illusions and hallucination [3]. Proposed factors leading to schizophrenia include maternal exposure to stress (prenatal environmental insults), infection and/or immune activation, nutritional deficiencies, obstetric complications [4] and use of cannabis [5]. Changes in dopamine neurotransmission in schizophrenia have been related to hallucinations and delusions in the patients. Effective therapy which blocks the dopamine system resolves these two clinical conditions [6].

Nitric oxide (NO) is a molecule formed in the cells through the conversion of the amino acid, L-arginine to NO by the action of nitric oxide synthase (NOs) [7]. NO participates in other biological processes such as vasodilatation, bronchodialation, inhibition of phagocyte, platelets aggregation, regulation of blood pressure, and defense against invading pathogens [8,9]. The synergistic effects of impaired synthesis, exhaustion of NO during anti-oxidative activities and diversion of the NO to the peroxynitrite (ONOO) pathway could contribute to its deregulated levels in certain disease conditions [10-12]. The vital roles played by NO in neurotransmission and maintenance of memory make it an interesting target molecule for schizophrenia research.

Hydrogen peroxide, a pro-oxidant generated at physiological level in the apical pole of the thyroid cells is required by normal thyroid gland to oxidize iodide into iodine in a reaction catalyzed by thyroperoxidase [13-15]. Other reactions catalyzed in thyroid hormogenesis include the coupling of mono-iodotyrosine and di-iodotyrosine, and in the synthesis of reverse T3 [16]. Chittiprol et al [17] reported significantly higher level of oxidative stress and lipid peroxidation which decline with treatment in schizophrenic patients. Yazici et al [18] also reported significantly higher levels of thyroid hormones in schizophrenia. None of the previous researchers related the abnormal patterns of thyroid hormones in schizophrenics to excessive free radical generation. Since hydrogen peroxide catalyses a series of chemical reactions in the synthesis of thyroid hormones, it may be possible to have free radical induced hyperthyroidism in schizophrenia. This study was designed to probe further in schizophrenic research, and bridge other gaps by determining the levels of thyroid hormones and makers of oxidative stress in schizophrenics.

## Materials and methods

### Materials

Thirty (17 males and 13 females) newly diagnosed patients with acute schizophrenia (duration- between 2.5 and 4.0 months), attending Psychiatric Hospital Abeokuta, Nigeria and Psychiatric Hospital Yaba, Lagos, Nigeria were recruited for this study before initiation of chemotherapy. The patients were traders and middle class civil servants. Informed consent was obtained from their relations before being included in this study. The patients were residents of Ogun and Lagos states in Nigeria. DSM-IV diagnosis of schizophrenia was established by the consultant psychiatrist in charge of the schizophrenic patients, using the Assessment of Positive and Negative Syndrome Scale (PANSS) [19]. The schizophrenic patients were either receiving spiritual treatment or not under specific treatment before being recruited for this study. Those patients with history of hard drugs (including alcohol and cigarette), pre-diagnosis medications (e.g. antiparkinsonian/antipsychotic drugs), chronic infections, liver disease and diabetes mellitus were excluded from this study. Twenty five (13 males and 12 females) apparently healthy staff of Olabisi Onabanjo University Teaching Hospital, Shagamu, Ogun state, Nigeria, served as controls. None of the controls was on any medication (including alcohol, cigarette and multivitamins), had history of chronic infections, malnutrition syndrome, depression, psychosis or metabolic dysfunction (such as diabetes mellitus, liver diseases, cancer) that could interfere with their oxidative metabolites and thyroid hormone status. The experimental protocol was approved by the Research Ethical Committee of Psychiatric Hospital Abeokuta, Nigeria.

5 ml of venous blood sample was taken from the anticubital vein of each participant. The sample in lithium heparin bottle was centrifuged within one hour of collection, after which the plasma was separated and stored at -70°C until assayed.

### Methods

T4, T3 and TSH were determined by using commercially prepared enzyme linked immunosorbent assay (ELISA) reagents (cat. numbers Z01208, Z01232 and Z01237 respectively) by Dialab, Gesellschaft, Vienna, as described by Young et al [20]. The MDA, a product of lipid peroxidation was determined by using the method of Varshney and Kale [21]. TAP was determined using the ferric reducing/antioxidant power (FRAP) assay [22,23]. Method of Harma et al [23] was used for the determination of TPP. The OSI, an indicator of the degree of oxidative stress was determined as the percent ratio of the total plasma peroxide (μMol H_2_O_2_/L) to the total antioxidant activity (μmol.Trolox equiv./L) [23]. NO was determined using the method described by Wanchu et al [24].

### Statistical analysis

Data processing and statistics were done using SPSS version 10. The data were expressed as Mean ± SD. Student (t) test was used for comparison of schizophrenics and controls. Pearsonian correlation coefficient (r) was calculated. The changes were considered significant, when p-values were less than 0.05.

## Findings

Age and gender distributions in the schizophrenics and controls were expressed in Table 1. The schizophrenics and the controls were age and sex matched. The results of this study indicate significantly (p < 0.01) increased plasma levels of TPP, OSI and MDA in schizophrenics when compared with the controls. Meanwhile, the plasma levels of NO and TAP decreased significantly (p < 0.01) in schizophrenia when compared with the controls (Table 2). This is an indication of oxidative stress in the schizophrenics. The plasma levels of T3 and T4 (Table 3) increased significantly (p < 0.01 and p < 0.05 respectively) while the mean value of TSH was significantly lower (p < 0.01) in the schizophrenic patients when compared with the controls. There were positive correlations observed between T3 and MDA (p <**0.05**) and between T3 and TPP (p <**0.01**) in the schizophrenics (Tables 4 and 5 respectively), showing strong association between oxidative stress and T3 synthesis.

**Table 1:** Physical Characteristics of Schizophrenics and Controls

Characteristics	Schizophrenics (N = 30)	Controls (N = 25)
Age (years)	25-43	22-45
**Gender:**		
Male (n)	17	13
Females (n)	13	12.

N = total number of subjects used for the study

n = gender number in the study

**Table 2:** Markers of Oxidative Stress in Schizophrenics and Controls

	Schizophrenics	Controls	p values
MDA (nMol/ml)	9.5 ± 3.0	6.5 ± 1.9	< 0.01*
TPP (μMol H_2_O_2_/L)	16.5 ± 1.2	10.1 ± 0.5	< 0.01*
TAP (μMol Trolox equiv./L)	975 ± 140	1300 ± 196	< 0.01*
OSI (%)	1.7 ± 0.8	0.8 ± 0.3	< 0.01*
NO (μMol/L)	17.8 ± 6.4	24.7 ± 5.5	< 0.01*
N	30	25.	

MDA = malondialdehyde. OSI = oxidative stress index

TPP total plasma peroxides. NO = nitric oxide

TAP = total antioxidant potential. N = number of subjects used in the study

* = significantly different from controls

**Table 3:** Thyroid Hormones Levels in Schizophrenics and Controls

	Schizophrenics	Controls	p -values
T3 (ng/ml)	7.5 ± 1.5	1.4 ± 0.5	< 0.01*
T4 (nmol/L)	65.0 ± 18.9	50.4 ± 12.7	< 0.05*
TSH (miU/L)	0.25 ± 0.2	1.4 ± 0.5	< 0.01*
N	30	25.	

T3 = tri-iodothyronine TSH = thyroid stimulating hormone T4 = thyroxine

N = number of subjects used in the study.

* = significantly different from controls

**Table 4:** Correlation between MDA and thyroid hormones in schizophrenics (N = 30)

Group	Correlation coefficient (r)	p-values
MDA-T3	**0. 51**	< 0.05*
MDA-T4	**0.3**	**0.4**
MDA-TSH	**0.36**	**0.2**.

* = significantly different from controls

N = number of subjects used in the study

**Table 5:** Correlation between TPP and thyroid hormones in schizophrenics (N = 30)

Group	Correlation coefficient (r)	p-values
TPP-T3	**0.8**	< 0.01*
TPP-T4	**0.46**	**0.15**
TPP-TSH	**0.41**	**0.09**.

* = significantly different from controls

N = number of subjects used in the study

## Discussion

Hydrogen peroxide has been identified as an important factor in thyroid hormogenesis by the previous researchers. Overproduction of this pro-oxidant/free radical has also been implicated in the pathogenesis of many disease conditions. Several reports showed that sources of free radicals in schizophrenics include auto-oxidation of cathecholamines, the trauma, post traumatic stress disorder, high neuronal activity, increased oxygen consumption during high neuronal activity [25], several chronic medical illnesses [26], somatic symptoms [27], malnutrition, endocrine disorder and infections [4]. Oxidative stress results when there is increased free radical generation beyond the detoxification capacity of the antioxidant defense system [28]. The possible causes of free radical generation in our schizophrenic patients could be malnutrition, high neuronal activity; chronic infections and trauma (in abandoned or roughly handled patients). Our results agree with previous researchers [29] who reported excessive free radical generation, lipid peroxidation and oxidative stress in schizophrenics. Evidences from previous researchers also indicate increased plasma lipid peroxidation and decreased levels of essential fatty acids in the brains of the schizophrenic patients [30,31].

Total antioxidants potential (TAP) is an index of all classes of antioxidants. Increased demand in the detoxification/neutralization process of free radicals or micronutrient deficiency (malnutrition) may be responsible for the lower levels of antioxidants in the schizophrenics [28]. This increased utilization of antioxidants demonstrated in schizophrenics leads to depressed plasma levels and reduced urinary excretion of ascorbic acid after an ascorbic acid load [32,33]. Our findings agree with a number of reports which indicate significantly lower level of total antioxidant in schizophrenics. Dadheech et al [28] reported significantly lower levels of superoxide dismutase and glutathione peroxidase in schizophrenic patients. In a study by Raffa et al [29], significantly lower levels of superoxide dismutase and catalase were also reported in schizophrenic patients. In their study, the antioxidant deficiency was associated with oxidative stress and malnutrition. This oxidative stress has also been associated with the aging process and chronic stage of the illness in some schizophrenics investigated by Dadheech et al [28].

The ameliorative effects of antioxidant vitamins C and E in some Wistar rats with ischemic brain injury confirmed the deteriorative effect of the oxidative stress in the brain [17]. In another study, decreased plasma level of the cell membrane antioxidant, α-tocopherol was associated with increased oxidative stress in schizophrenics [34]. An increased plasma level of histamine is a factor in the behavioral defect commonly observed in schizophrenia [35]. In-vitro studies showed that vitamin C reduces histamine levels by promoting the formation of a mono-oxygenated form of N-acetylhistamine [36]. But contrary to these opinions, Straw et al [37] reported that there was neither a change in psychopathology of schizophrenic patients nor was there any apparent pharmacokinetic interaction with haloperidol after ascorbic load.

Nitric oxide is produced in the endothelia of the blood vessels from the amino acid, L-arginine by the enzymatic reaction with oxygen catalyzed by nitric oxide synthase, in the presence of NADPH, tetrahydrobiopterin and flavin adenine nucleotides as co-factors. The highest concentration of NO is found in the brain for the purpose of cell migration, formation of synapses, receptor mediated neurotransmission, cognitive abilities [38], maintenance of memory, and for regulation of blood pressure [7]. Brain NO is involved in various processes such as immunological responses, neuroplasticity, neurodevelopment and neurotransmitter release. The reaction between peroxides and NO in a pathway leads to the production of peroxy-nitrite [11,12,16,39]. Peroxy-nitrite has greater ability to diffuse away from the site of production and can induce more selective and ultimately harmful oxidative damage such as the initiation of lipid peroxidation [40,41]. Several research findings, ranging from genetic and post mortem to neurochemical and psychopharmacological evidence support an involvement of the NO-signaling system in schizophrenia pathophysiology. There have been conflicting reports on the levels of NO in schizophrenia: some of them suggesting an increase of the NO-mediated neurotransmission, and another part supporting a decrease [42]. Contrary to the report of Zoroglu et al [43] who demonstrated significantly higher level of NO and adrenomedullin in schizophrenia, our result shows significantly lower level of NO in acute schizophrenia. Diversion of the nitric oxide to the peroxy-nitrite pathway mediated by high level of peroxides (i.e. TPP) could cause the lower plasma level of NO in our schizophrenic patients. Since L-arginine is the precursor of NO, inadequate intake of L-arginine (malnutrition) could also cause impaired synthesis and lower level of NO in these schizophrenic patients. This finding therefore calls for further studies to establish the levels of peroxy-nitrite and NO in the brain and plasma of schizophrenic patients.

To the knowledge of the authors, this study is the first to relate the plasma markers of oxidative stress with thyroid hormogenesis in schizophrenics. In the synthesis of thyroid hormones, hydrogen peroxide is required by the thyroid cells to oxidize iodide into iodine in a reaction catalyzed by thyroperoxidase [13-15] and also in the coupling of mono-iodotyrosine and di-iodotyrosine, and synthesis of reverse T3 [16]. The fact that higher levels of total plasma peroxides and MDA correlate with T3 in schizophrenics is the novel of this study. Our result agrees with the reports of the previous researchers [44] that significantly higher levels of free T3 and free T4 are features of schizophrenia. Yazici et al [18] also reported significantly higher levels of total T3 and free T3 in schizophrenics when compared with the controls. Baumgartner et al [45] also observed abnormal T4 level in acute schizophrenic patients, but the serum level declined in treated schizophrenic patients when compared with the control.

In conclusion, oxidative stress in schizophrenics may enhance thyroid hormone synthesis. Therefore, adjuvant antioxidant therapy may be a novel approach in the treatment of schizophrenic patients to avert the consequences of oxidative stress.

## Competing interests

The authors declare that they have no competing interests.

## Authors' contributions

AMO, OAO and EOO designed the research, AMO did the analysis and all authors contributed and approved the final manuscript.

## Limitations of the study

Non-compliance of relations of many patients and limited numbers of acute cases of schizophrenia without pre-diagnosis medications contributed to the small sample size.
